# *PpNAC187* Enhances Lignin Synthesis in ‘Whangkeumbae’ Pear (*Pyrus pyrifolia*) ‘Hard-End’ Fruit

**DOI:** 10.3390/molecules24234338

**Published:** 2019-11-27

**Authors:** Mingtong Li, Chenxia Cheng, Xinfu Zhang, Suping Zhou, Caihong Wang, Chunhui Ma, Shaolan Yang

**Affiliations:** 1 College of Horticulture, Qingdao Agricultural University, No. 700 Changcheng Road, Chengyang, Qingdao 266109, China; limingtongqau@126.com (M.L.); chengchenxia@163.com (C.C.); zxftea@163.com (X.Z.); chwang@qau.edu.cn (C.W.); machunhui2000@163.com (C.M.); 2 Department of Agricultural and Environmental Sciences, College of Agriculture, Tennessee State University, 3500 John Merritt Blvd, Nashville, TN 37209, USA; ZSUPING@Tnstate.edu

**Keywords:** ‘Whangkeumbae’, pear, hard-end, *NAC*, lignification

## Abstract

A disorder in pears that is known as ‘hard-end’ fruit affects the appearance, edible quality, and market value of pear fruit. RNA-Seq was carried out on the calyx end of ‘Whangkeumbae’ pear fruit with and without the hard-end symptom to explore the mechanism underlying the formation of hard-end. The results indicated that the genes in the phenylpropanoid pathway affecting lignification were up-regulated in hard-end fruit. An analysis of differentially expressed genes (DEGs) identified three NAC transcription factors, and RT-qPCR analysis of *PpNAC138*, *PpNAC186*, and *PpNAC187* confirmed that *PpNAC187* gene expression was correlated with the hard-end disorder in pear fruit. A transient increase in *PpNAC187* was observed in the calyx end of ‘Whangkeumbae’ fruit when they began to exhibit hard-end symptom. Concomitantly, the higher level of *PpCCR* and *PpCOMT* transcripts was observed, which are the key genes in lignin biosynthesis. Notably, lignin content in the stem and leaf tissues of transgenic tobacco overexpressing *PpNAC187* was significantly higher than in the control plants that were transformed with an empty vector. Furthermore, transgenic tobacco overexpressing *PpNAC187* had a larger number of xylem vessel elements. The results of this study confirmed that *PpNAC187* functions in inducing lignification in pear fruit during the development of the hard-end disorder.

## 1. Introduction

The hard-end disorder in pear fruit occurs in many pear-growing regions [1,2]. The disease is frequently found in the United States of America (USA) in pear varieties, such as ‘Anjou’ (*Pyrus communis L*), ‘Winter Nelis’ (*P. dimorphophylla*), and ‘Comice’ (*P. communis*) [3,4]. In recent years, the hard-end disorder also appeared in some Asian pear varieties, including ‘Whangkeumbae’ (*P. pyrifolia*) and ‘Xueqing’ (*P. nivalis*), and other varieties may also be affected [5,6]. Hard-end of pear is a physiological disorder. Pear varieties that are grafted on Japanese pear (*P. serotina Rehd*) rootstocks often exhibit this disorder due to scion-rootstock compatibility problems that cause water imbalance problems and low Ca content and a low Ca/K ratio [6,7,8]. Fruits with a hard-end are culled out during grading and packing, and severely misshaped fruits are deemed as unmarketable. The first symptom of hard-end disorder is observed as an abnormally green or yellow color at the blossom end when the fruits have grown to one-third or half of their full size. A readily apparent protrusion of the calyx forms due to the delayed development of the surrounding tissues. The epidermis over the calyx-end of the fruit appears to be tight and shiny, and the flesh near the calyx turns dry and hard due to an accumulation of lignin [5].

We previously reported that lignin content and the number of stone cells significantly increases during hard-end development in ‘Whangkeumbae’ fruit [5]. These hard-end pear fruit also contain high levels of enzymes that are involved in lignin synthesis, including phenylalanine ammonia lyase (PAL), 4-coumarate: coenzyme A ligase (4CL), cinnamyl alcohol dehydrogenase (CAD), and peroxidase (POD). As a result, the development of hard-end in pear fruit is correlated with lignin accumulation. Lignin is a phenylpropanoid-derived polymer that is deposited in secondary cell walls to increase the mechanical strength of xylem tissues in vascular plants and provide defense against attacks from pathogens [9]. Several genes and transcription factors are known to be involved in lignin biosynthesis, including PAL, 4CL, CAD, cinnamoyl CoA reductase (CCR), and caffeic acid 3-O-methyltransferase (COMT) [10,11]. In pear fruit, the transcript levels of *Pp4CL1*, *PpCAD1*, and *PpCAD2* were elevated in hard-end pear, relative to normal fruit [5].

Several transcription factors that are involved in lignin biosynthesis, such as MYB (myeloblastosis), NAC (NAM, ATAF, and CUC), bHLH (basic helix-loop-helix), and others, have been identified and characterized in *Arabidopsis thaliana*, tobacco (*Nicotiana tabacum*), and loquat (*Eriobotrya japonica*) fruit [12,13,14,15]. The NAC transcription factor family is one of the largest families of plant-specific transcription factors and they participate in several physiological processes [16]. Most proteins that contain a NAC domain are located at the upstream end of a regulatory network. In *Arabidopsis thaliana*, *AtNST1* and *AtNST2* promote secondary wall thickening in the endothecium of anthers, and knock-out mutants of *NST1* and *NST3* lose secondary-wall deposition in stems [17]. Several studies have reported that the NAC genes function as master switches in the biosynthetic pathways for cellulose, xylan, and lignin by initiating a transcriptional signaling network that either affects MYB transcription factors or regulates the expression of structural genes [18,19]. A study on loquat chilling-induced lignification demonstrated that EjNAC3-regulated the expression of the *EjCAD-like* gene, which is a key gene in lignification [20].

‘Whangkeumbae’ pear fruit was previously reported to accumulate lignin during the development of hard-end fruit. In the present study, DEGs were identified during hard-end disorder in comparison to the normal fruits in pears. RNA-seq was performed and it led to the identification of NAC transcription factors that potentially control secondary cell wall and lignin deposition during hard-end disorder, and then continue with the characterization of such gene(s).

## 2. Results

### 2.1. RNA-Seq Analysis

**Figure 1:**
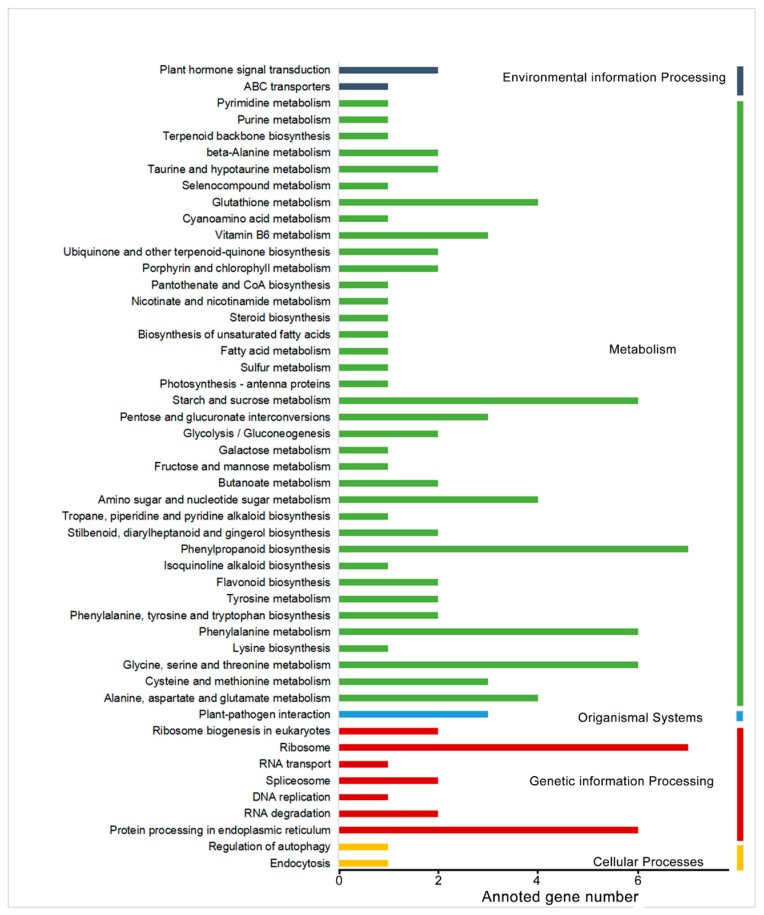
Kyoto Encyclopedia of Genes and Genomes (KEGG) enrichment analysis and the annotation of DEGs in control vs. hard-end fruit. The yellow columns indicate cellular processes, the red columns indicate genetic information processing, the blue columns indicate organismal systems, the green columns indicate metabolism, and the purple columns indicate environmental information processing. The figure displays the number of annotated genes in each category.

**Figure 2:**
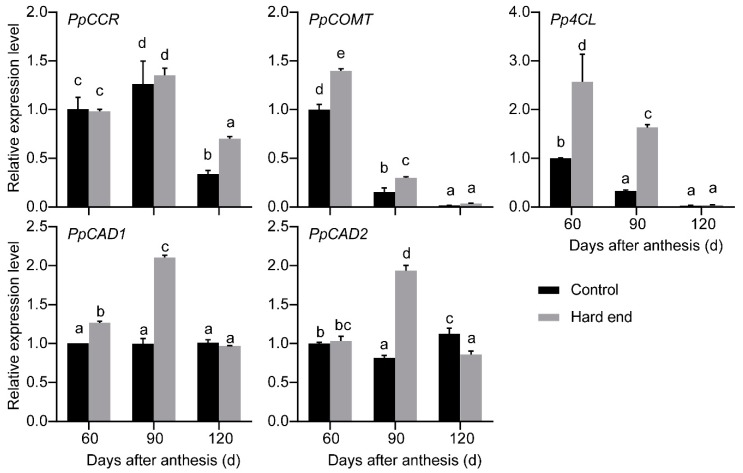
The relative expression of lignification-related genes during the period of fruit development. a–e, the different letters indicate significant differences between control and hard end fruit (*p* < 0.05; Duncan’s multiple-range test).

A total of 545 DEGs were identified in the comparison between hard-end and normal fruit in samples that were collected on the day of harvest (120 d after anthesis). A KEGG pathway enrichment analysis placed these DEGs into six pathways, which include protein processing in endoplasmic reticulum; ribosome; glycine, serine, and threonine metabolism; phenylalanine metabolism; phenylpropanoid biosynthesis; and, starch and sucrose metabolism (Figure 1). The phenylpropanoid biosynthesis and ribosome were enriched in seven genes, which was the highest among all of the identified pathways. The DEGs in the phenylpropanoid biosynthesis pathway were annotated as *PpCCR*, *PpC3H*, *PpF5H*, *Pp4CL*, *PpCOMT*, *PpPOD* (GDR accession No., PCP040222, PCP022543, PCP016311, PCP044725, PCP024172, PCP007841, PCP013947, and PCP030808). In our previous research, we found that the sclereid content of hard end fruit was greater with critical lignification when compared to that of the control fruit during the fruit development (from 60 days before harvest to the day at harvest) [5]. We conducted qPCR at 60, 90, and 120 days after anthesis to evaluate the expression patterns of these genes related to lignin during fruit development. Lignin-related genes *PpCCR*, *Pp4CL*, *PpCOMT*, *PpCAD1*, and *PpCAD2* also exhibited significantly higher levels of expression in hard-end fruit, relative to normal pear fruit (Figure 2).

### 2.2. The Phylogenetic Analysis of PpNACs and Their Expression Pattern

**Figure 3:**
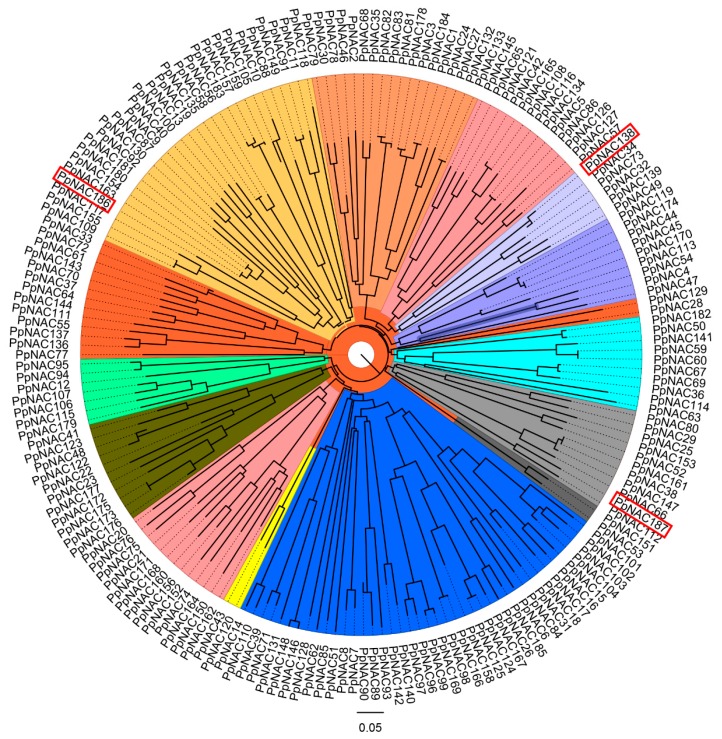
Phylogenetic analysis of pear NAC genes based on deduced amino acid sequences. *PpNAC138*, *PpNAC186*, and *PpNAC187* are framed in a red box. Genes listed with the same color indicate genes that are highly homologous.

Transcription factor family genes were among the genes that were represented in the transcriptome data. NAC transcription factor genes were selected from the identified transcription factors for further analysis. Three *PpNAC* genes of PCP044783, PCP012487, and PCP013078 were named as *PpNAC138*, *PpNAC186*, and *PpNAC187*, according to the reference (Figure 3) [21]. The three *NAC* genes that were identified as DEGs, *PpNAC187* was up-regulated, *PpNAC138* and *PpNAC186* were down-regulated (Log2FC > 2 or Log2FC < −2) in hard-end fruit, relative to normal pear fruit. 

**Figure 4:**
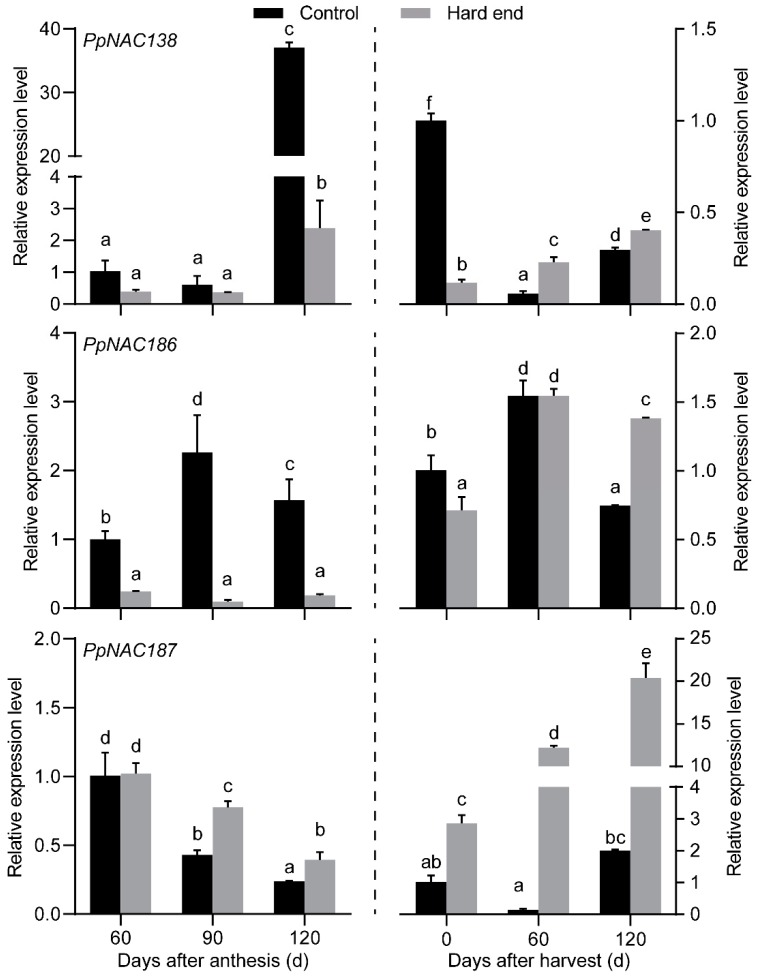
The relative expression of NAC transcription factors during fruit development and postharvest storage. a-f, the different letters indicate significant differences between control and hard end fruit (*p* < 0.05; Duncan’s multiple-range test).

In our previous work, we found that the firmness in the hard end fruit increased from 60 days after harvest and then reduced at 120 days after harvest [5]. RT-qPCR analyzed the expression patterns of *PpNAC138*, *PpNAC186*, and *PpNAC187* in hard-end and normal fruit during fruit development and postharvest storage. The results indicated that the relative transcript abundance of *PpNAC187* exhibited a significant increase of expression in hard-end fruit at 90 and 120 d after anthesis, while *PpNAC138* and *PpNAC186* were all down-regulated in hard-end fruits during fruit development (Figure 4). The relative transcript abundance of *PpNAC187* gradually increased in hard-end fruit during postharvest storage, while no significant changes in expression were detected in normal fruit. The transcript abundance of *PpNAC187* was consistently higher in the hard-end fruit than in normal fruit. The relative abundance of *PpNAC138* exhibited some greater level of expression in hard-end fruit than in normal fruit at 60 d, while *PpNAC186* only exhibited a higher level of expression at 120 d after harvest in the hard-end pear fruits (Figure 4). *PpNAC187* was selected for further analysis, as it exhibited the greatest difference in expression in hard-end vs. normal fruit.

### 2.3. Subcellular Localization of PpNAC187

**Figure 5:**
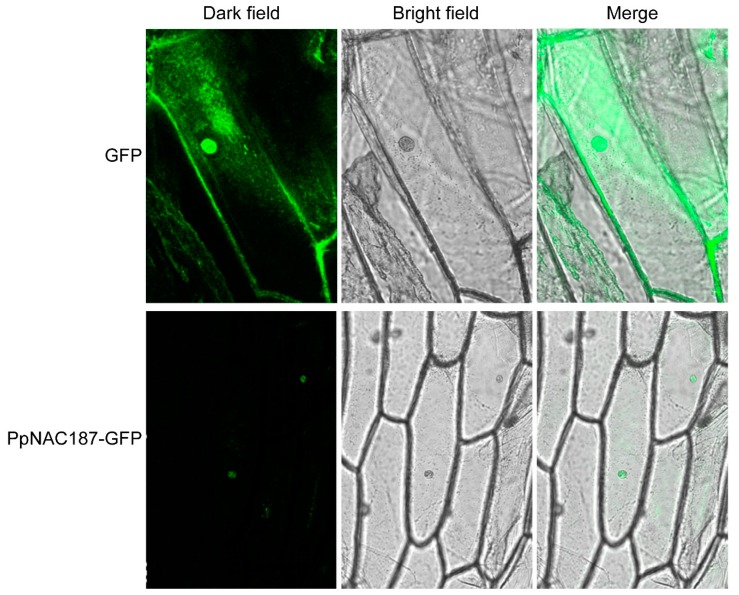
Subcellular localization of a PpNAC187-GFP fusion protein in onion epidermal cells. The Green Fluorescent Protein (GFP) protein was C-terminal to PpNAC187. The laser-scanning confocal microscopy was used to obtain light, fluorescent, and merged images. Dark images represent fluorescent images, bright represent light images, and merged represent merged dark and light images.

A pCambia1300-PpNAC187 vector, carrying a Green Fluorescent Protein (GFP) reporter protein, was constructed and then inoculated into onion scales to determine the subcellular localization of *PpNAC187*. An empty vector was used as a control. When viewed under a fluorescent microscope, the cytomembrane and nucleus in living onion epidermal cells that were infected with the pCambia1300 empty vector exhibited green fluorescence. In contrast, only the nucleus exhibited green fluorescence in onion epidermal cells that were infected with the pCambia1300-PpNAC187. These results indicate that the *PpNAC187* transcription factor is expressed and localized in the nucleus of onion epidermal cells (Figure 5).

### 2.4. Transient Expression of PpNAC187 in ‘Whangkeumbae’ Pear Flesh

**Figure 6:**
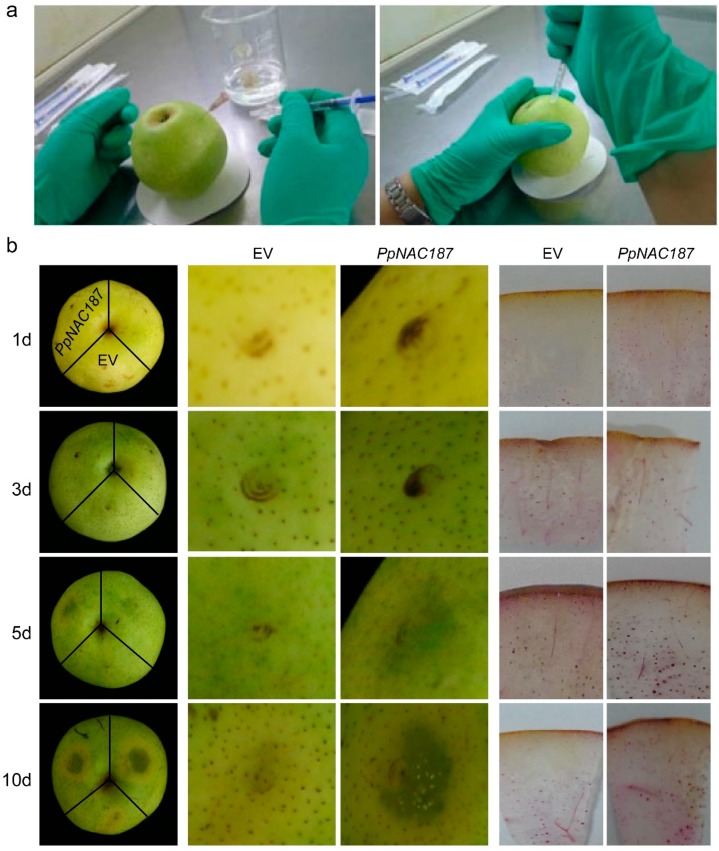
Transient expression of *PpNAC187* in ‘Whangkeumbae’ pear fruit. (**a**) Infiltration of the pSuper1300-PpNAC187 or pSuper1300 empty vector into ‘Whangkeumbae’ pear fruit. (**b**) Fruit phenotype (left) and Wiesner staining of fruit sections (right) at 1 d, 3 d, 5 d, and 10 d after infiltration of the vectors. EV represents images of fruit infiltrated with the pSuper1300 empty vector, and *PpNAC187* represents images of fruit infiltrated with the pSuper1300-PpNAC187 vector.

Transient expression analysis of *PpNAC187* was conducted in ‘Whangkeumbae’ pear flesh by injecting pSuper1300-PpNAC187 into fruit flesh, while the injection of an empty vector served as a control (Figure 6a). No obvious changes in the pear fruit surface were observed over a three-day period following injection with empty vector of all the fruit. However, subsequently, the color around the inoculation site of fruit injected with *Agrobacterium* harboring the pSuper1300-PpNAC187 vector changed to dark green at 5 d after inoculation, and the green color progressively deepened by the 10th d after inoculation. In contrast, the control fruit that was inoculated with the empty vector exhibited no significant change in color over the ten-day post-injection period. The results of the lignin staining indicated no obvious differences between fruit inoculated with empty vector vs. pSuper1300-PpNAC187 after 3 d, but the level of staining was noticeably higher in pSuper1300-PpNAC187 inoculated fruit than control fruit at 5 d and 10 d post-injection (Figure 6b). The results on the transient expression of *PpNAC187* are in agreement with the increased lignin accumulation that was observed in ‘Whangkeumbae’ hard-end fruit. 

**Figure 7:**
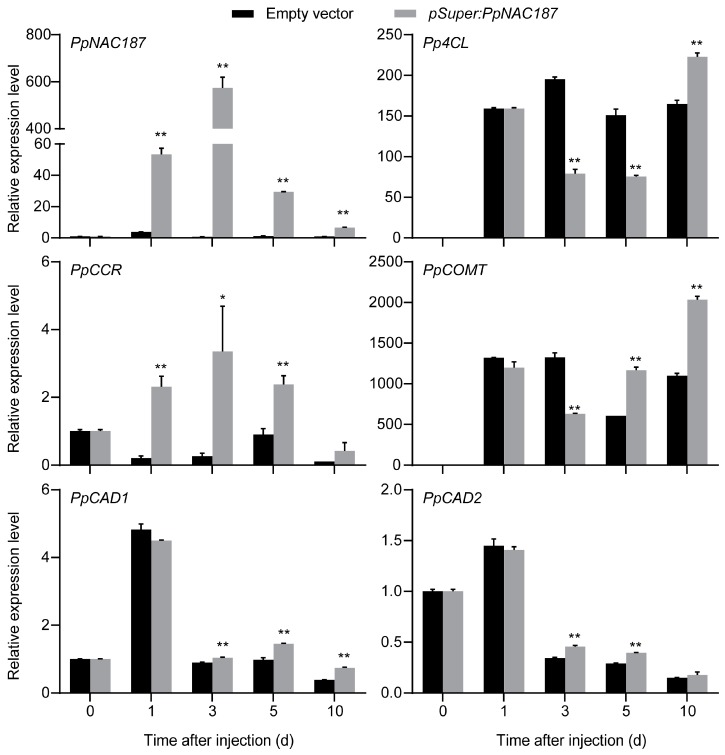
The relative expression of lignification-related genes in pear fruit surrounding the site of infiltration. 35S:PpNAC187 represent ‘Whangkeumbae’ pear fruit in which *PpNAC187* was transiently expressed. Empty vector represent fruit that were infiltrated with the pSuper1300 empty vector. The x-axis represents time after infiltration, and the y-axis represents relative expression. Asterisks indicate significant differences between empty vector and 35S:PpNAC187 fruit (*, *p* < 0.05; **, *p* < 0.01; two-tailed *t*-test).

The expression of *PpNAC187* and lignin-synthesis-related genes was also analyzed in fruit tissues surrounding the injection site. In comparison to fruit that were injected with the empty vector control, the relative expression level of *PpNAC187* was higher in the fruit injected with the pSuper1300-PpNAC187 vector. The expression pattern of *PpCCR* was analogous to *PpNAC187*, which exhibited an increase in expression at 3 d post-injection. The expression levels of *Pp4CL* and *PpCOMT* increased after 10 d and 5 d post-injection, respectively. There was a significant increase in both *PpCAD1* and *PpCAD2* expression of fruit that were injected with the pSuper1300-PpNAC187 vector when compared to empty vector after 3 d post-injection (Figure 7). Thus, it was concluded that the expression of *PpNAC187* (TF) and *PpCCR*, *PpCOMT*, *PpCAD1*, and *PpCAD2* (lignin biosynthesis genes) was correlated with the lignification of flesh tissues in pears and reflected what occurred during the normal development of hard-end pear fruit.

### 2.5. Functional Verification of PpNAC187 in Transgenic Tobacco

**Figure 8:**
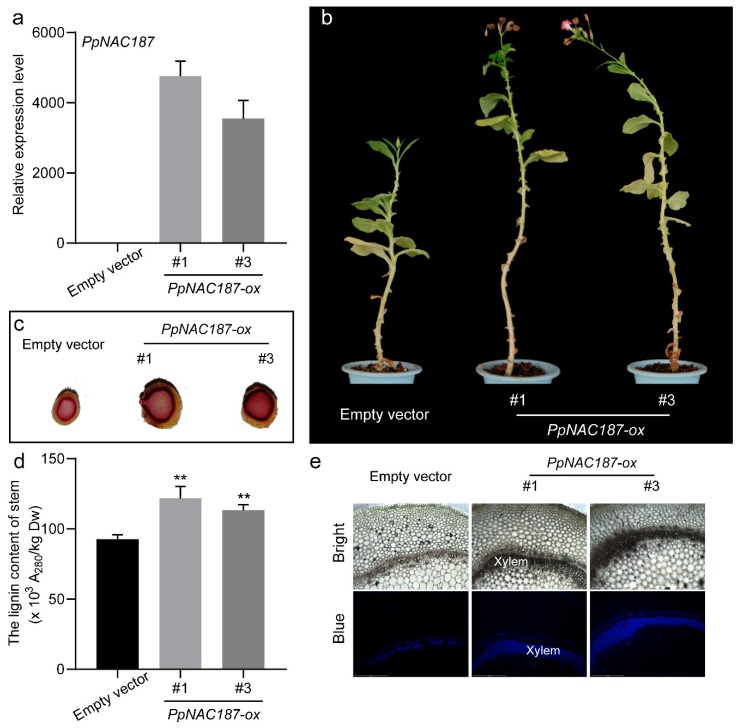
*PpNAC187* increase the lignin content in the stem of transgenic tobacco plant. (**a**) The relative expression level of *PpNAC187* in transgenic tobacco. (**b**) Phenotype of empty vector and *PpNAC187*-overexpressing (*PpNAC187*-ox) transgenic tobacco lines. (**c**) Transverse sections of stem were stained with phloroglucinol–HCl for detection of lignin. (**d**) Lignin content in transgenic tobacco stem tissues. (**e**) Autofluorescence of the stem transverse slice. Bright: bright field images, Blue: blue autofluorescence. #1 and #3 means two independent *PpNAC187* transgenic lines. Significant differences between the empty vector and *PpNAC187*-ox plants are indicated (**, *p* < 0.01; two-tailed *t*-test).

**Figure 9:**
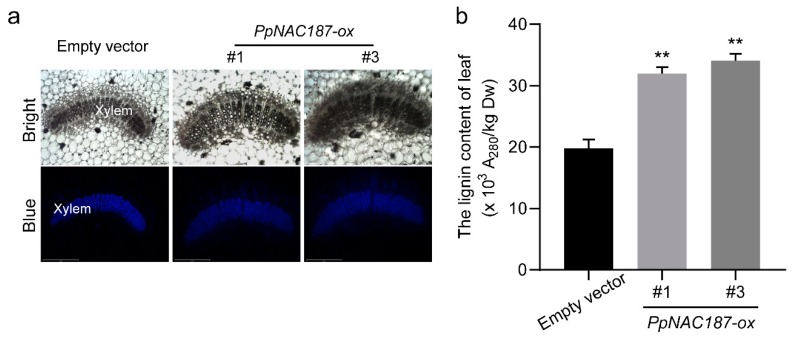
*PpNAC187* increase the lignin content in the leaves of transgenic tobacco plants. (**a**) Autofluorescence of transverse slice in the leaf veins. Bright: bright field images, Blue: blue autofluorescence. (**b**) Lignin content of leaves in the empty vector and *PpNAC187*-ox plants. #1 and #3 means two independent *PpNAC187* transgenic lines. Significant differences between the empty vector and *PpNAC187*-ox plants are indicated (**, *p* < 0.01; two-tailed *t*-test).

Transgenic tobacco plants overexpressing *PpNAC187* were generated while using an *Agrobacterium*-mediated transformation method. Insertion of *PpNAC187* into the tobacco genome was confirmed by PCR analysis. Two independent *PpNAC187* transgenic lines (#1 and #3) were selected. *PpNAC187* was highly-expressed in #1 and #3 *PpNAC187*-overexpressing transgenic plants and it was not expressed at the empty vector line (Figure 8a,b). The level of stem’s lignin staining was higher in *PpNAC187*-overexpressing lines than that in empty vector plants (Figure 8c). The lignin content in stem tissues of *PpNAC187*-overexpressing lines was notably higher than in empty vector line (Figure 8d). The autofluorescence within the stem sections in *PpNAC187*-overexpressing plants was also more pronounced when compared to the control tobacco plants that were transformed with an empty vector (Figure 8e). The autofluorescence within the leaf veins in *PpNAC187*-overexpressing plants was more pronounced than the empty vector line (Figure 9a). Meanwhile, the lignin content in leaf tissues of *PpNAC187*-overexpressing lines was also higher than that in empty vector line (Figure 9b). *PpNAC187*-overexpressing plants also grew more fibrous roots relative to the empty vector line (Figure S1a). There was no apparent difference in the lignin content in root tissues between *PpNAC187*-overexpressing and empty vector plants (Figure S1b).

## 3. Discussion

The hard-end disorder of ‘Whangkeumbae’ fruit is a major problem in the pear industry. Hard-end fruits contain significantly more and larger sclerotic cells in the calyx-end of the fruit when compared to normal pear fruit, as well as a higher level of synthesis and deposition of lignin [5]. Several genes, including *PAL*, *4CL*, *CCR*, *COMT*, and *CAD*, are components of the phenylpropanoid pathway that are associated with lignin synthesis [22,23,24,25,26]. Among these genes, *PpCAD1* and *PpCAD2* are continuously expressed at high levels during fruit development in fruit exhibiting hard-end symptoms [5]. The *CCR* and *CAD* family genes are also responsible for the regulation of lignin synthesis and stone cell development in pear fruit [10]. In the present study, KEGG analysis of transcriptome data from hard-end and normal ‘Whangkeumbae’ pears revealed several DEGs that are part of the phenylpropanoid biosynthesis pathway. Based on RPKM values, the expression level of these genes was notably higher in the hard-end fruit than in normal fruit. These results indicate that the lignin synthesis pathway is more highly-activated in hard-end fruit than in normal ‘Whangkeumbae’ pear fruit, which is in accordance with our previous research showing lignin accumulation during the development of hard-end symptoms [5].

Many physiological activities of plants are regulated by the activity of transcription factors [27]. Previous studies have reported that NAC transcription factors are involved in lignin synthesis in fruits. In loquat fruits, *EjNAC1* expression was induced in response to low temperature, but it was inhibited by a heat treatment (HT), the latter of which also inhibited lignification [15]. In the present study, the conducted RNA-seq analysis revealed that three pear *NAC* genes are expressed at significantly different levels in hard-end ‘Whangkeumbae’ pear fruit, relative to normal pear fruit. In particular, the expression level of *PpNAC187* in hard-end fruit was significantly higher than that in normal fruit during the development, as well as postharvest storage. *PpNAC187* was localized in nuclei, which suggested that *PpNAC187* is a functional transcription factor. These results suggest that *PpNAC187* might be involved hard-end syndrome in pear fruit. When a vector containing *PpNAC187* was injected into pear flesh tissues, the relative expression level of *PpNAC187* was significantly enhanced, being concurrent with the lignin biosynthesis-related genes (*PpCCR*, *PpCOMT*, *PpCAD1*, and *PpCAD2*, Figure 7). We suggest that lignin synthesis is potentially influenced by the NAC transcription factor. Several NAC genes, including *AtVND* and *AtNST*, have been previously reported to be involved in the regulation of phenylpropanoid biosynthesis and these NAC TFs also play a role in secondary xylem development and/or secondary wall formation in *A. thaliana* [7,28,29]. Our previous studies demonstrated that lignin biosynthesis-related gene *PpCAD2* was involved in regulating the formation of the xylem vessel. Moreover, lignin content was significantly higher in the stem and leaf tissues of *PpNAC187*-overexpressing transgenic tobacco. Collectively, the data indicate that *PpNAC187* plays a role in enhancing lignin accumulation by inducing the expression of *PpCCR* and *PpCOMT* in ‘Whangkeumbae’ pear fruit during the development of hard-end symptoms. The ectopic expression of NACs in *PpNAC187*-overexpressing transgenic tobacco activated biochemical and metabolic processes, which resulted in a greater number of vessel elements, sclereids, and a higher level of lignin accumulation.

## 4. Materials and Methods

### 4.1. Plant Material

‘Whangkeumbae’ pear fruit were picked in orchards that were located in Wulong village of Laiyang city, Shandong province, People’s Republic of China. Hard-end fruit were picked from ten-year-old ‘Whangkeumbae’ pear trees in one orchard, and normal pears were harvested from healthy trees in another orchard. The normal and hard-end fruit were sampled at 60, 90, and 120 days after anthesis, and then sampled again at 0, 60, and 120 days after harvest when stored under 0 °C. Three biological replicates comprised of ten fruits each was used for each condition (normal vs. hard-end) and at each sampling timepoint. The fruit tissues that were near the bottom third of the calyx end were taken. After the removal of the peel and/or seed, fleshy tissues were sliced into small pieces (approximately 1 cm^3^) and then immediately frozen in liquid nitrogen. Samples were stored at −70 °C until further analysis.

### 4.2. RNA-Seq Analysis 

The calyx pulp of normal and hard-end fruit at 120 days after anthesis were RNA-seq analysis. Total RNA was extracted while using an RNA extraction kit (Omega, Doraville, GA, USA) according to the manufacturer’s instructions. The integrity and quality of the total RNA was evaluated while using a 2100 Bioanalyzer RNA Nano chip device (Agilent, Santa Clara, CA, USA). The poly A-mRNA fraction was enriched by treatment of the extracted RNA with oligo (dT) beads and it was then reverse-transcribed into first strand cDNA for use in the preparation of the sequencing libraries.

The cDNA libraries were sequenced while using an Illumina HiSeq 2500 system at the Biomarker Technologies Corporation (Beijing, China). The raw reads were first filtered to remove adaptors and low quality sequences and then mapped to the pear reference genome (https://www.rosaceae.org/species/pyrus/pyrus_communis/genome_v1.0) while using TopHat software [30]. A false discovery rate (FDR) < 0.01 and a fold change of ≥2 were used to identify the differentially expressed genes (DEGs). The predicted product of each unigene sequence was aligned to a set of proteins that were retrieved from the NCBI Nr, Swiss-Prot, Kyoto Encyclopedia of Genes and Genomes (KEGG), and Cluster of Orthologous Groups of proteins (COG) databases. The Reads Per Kb per Million Fragments (RPKM) was used to determine the expression level of genes. RPKM normalized the total number of reads for each unigene and gene length. The formula used to calculate was as follows: RPKM = total exon reads/(mapped reads (millions) × exon length (KB)). KEGG pathway enrichment analysis was performed while using KOBAS software and utilized an adjusted *p*-value of <0.05. The transcription factors were identified and classified into different families by reference to the NCBI Nr, Swiss-Prot, and COG databases. The raw sequences that were generated for ‘Whangkeumbae’ in this study were deposited in NCBI (NCBI BioProject Accession: SRP063324, http://www.ncbi.nlm.nih.gov/bioproject/PRJNA294723).

### 4.3. Reverse Transcription-Quantitative Polymerase Chain Reaction (RT-qPCR) 

Total RNA was extracted from pear flesh tissue while using RNA plant Reagent (TianGen, Shanghai, China), according to the manufacturer’s instructions. Tobacco leaf RNA was extracted while using an EASYspin Plant RNA Kit (Yuanpinghao, Beijing, China) and genomic DNA was removed by treatments with DNase (Fermentas, Vilnius, Lithuania). The cDNA was synthesized by reverse transcription using the Prime Script™ RT reagent Kit (Takara, Dalian, China), according to the manufacturer’s instructions and it was subsequently used as template in the RT-qPCR analyses. RT-qPCR was performed on a Light Cycler^®^ 480 instrument (Roche, Basel, Switzerland). The protocol included annealing at 94 °C for 5 min., followed by 40 cycles of 94 °C for 15 s, and 60 °C for 1 min. Actin genes from pear and tobacco were used for the normalization of transcript levels [31,32]. The gene-specific primers that were used in the RT-qPCR analyses were designed with Primer 3 (http://bioinfo.ut.ee/primer3-0.4.0/) software and they are listed in Table S1. Mean expression level was calculated while using the 2^−ΔΔCt^ method [33]. The expression level in normal fruit at 60 days after anthesis was set as 1 in the RT-qPCR analyses that were conducted on samples collected during fruit development, and the day of harvest was set as 1 in the post-harvest analyses. Three biological and three technical replicates were used in the RT-qPCR analysis of each gene at each timepoint. 

### 4.4. Cloning of PpNAC187

Total RNA isolation and cDNA synthesis followed the same protocol that was used in the RT-qPCR analyses. Table S2 shows the PCR primers used to clone PpNAC187. The PCR program was: 94 °C for 5 min, 35 cycles of 94 °C for 30 s, 60 °C for 1 min., and 72 °C for 1 min., followed by an extension cycle at 72 °C for 10 min. and a final cycle at 4 °C. The PCR products were cloned into PMD19-T vectors (Takara, Dalian, China). The open reading frame (ORF) of *PpNAC187* was amplified and cloned while using Phusion^®^ High-Fidelity DNA Polymerase (New England Biolabs, Beijing, China).

### 4.5. Sequence Alignment and Phylogenetic Analysis

The amino acid sequence alignment analysis of NACs was conducted while using Observed Divergency algorithm with DNAMAN software (version 4.0, Lynnon Biosoft Company, Foster, RI, USA). A phylogenetic tree was reconstructed with Figtree (http://tree.bio.ed.ac.uk/software/figtree/) online software (version 1.4.4, University of Edinburgh, Edinburgh, United Kingdom). The amino acid sequence alignment analysis of pear NACs were referred to Ahmad [21].

### 4.6. Construction of the Expression Vector

The ORF of *PpNAC187* was ligated into the expression vector, pCambia1300, under the control of a 35S promoter. The ORF fragment that was isolated by digestion with KpnI and HindⅢ was inserted into the expression vector, pSuper1300. The vectors, pCambia1300-PpNAC187 and pSuper1300-PpNAC187, were transferred into *Agrobacterium tumefaciens EHA105* while using the freeze-thaw method [34]. Table S2 lists the sequences of primers used to construct the expression vector.

### 4.7. Subcellular Localization of the PpNAC187 Transcription Factor

The subcellular localization of gene expression was determined while using the method that was described by Sun, with some modifications [35]. After incubation for 24 h at 28 °C in the dark, fresh onion scales (1.5 × 1 cm) were placed on a 9 cm plate with their inner surface submerged in a 10 mL *Agrobacterium* solution (OD_600_ = 0.6–0.8) supplemented with 20 mg acetosyringone/L for 15–20 min. The onion scales were then transferred to a 1/2 MS solid medium that was amended with 20 mg acetosyringone/L and cultured for 16–24 days at 28 °C. The onion scales were subsequently rinsed with water and the epidermal cell layers were peeled and directly transferred to glass slides. *Agrobacterium* harboring the pCambia1300-PpNAC187 or the empty pCambia1300 vector were used in the analysis of subcellular localization. The GFP of onion scales that were inoculated with these vectors were observed under a confocal laser scanning microscope (TCSSP5II, Leica, Weztlar, Germany).

### 4.8. Transient Expression of PpNAC187 in ‘Whangkeumbae’ Pear

The method of transient expression of *PpNAC187* in pear ‘Whangkeumbae’ followed the method that was described by Spolaore, with some modifications [36]. Holes were punched on the calyx end of hard-end fruits on the harvest day while using a sterile syringe needle. One ml of *Agrobacterium* solution (OD_600_ = 0.6–0.8) was then injected into the fruit via the holes using a syringe without a needle and the injected fruit was stored in the dark. Fruits that were inoculated with pSuper1300-PpNAC187 (treated) or the empty pSuper1300 vector (control) were photographed at the sampled timepoints. Samples were taken at 1, 3, 5, and 10 days after the injection, and they were immediately frozen in liquid nitrogen and then stored at −70 °C until further processing. Fifty fruits were used for each vector. Ten fruit for each vectors were randomly collected for the measurements at each time point.

### 4.9. Agrobacterium-Mediated Transformation of Tobacco with PpNAC187 

The empty pSuper1300 vector and pSuper1300-PpNAC187 were independently transformed into tobacco plants while using the *Agrobacterium*-mediated transformation method, as described by Zheng with some modifications [37]. Portions of tobacco leaves without veins were cut into discs (1 × 1 cm) and pre-cultured on MS solid medium for two days at 28 °C in the dark. The leaf discs were then submerged in 15 mL of *Agrobacterium* solution (OD_600_ = 0.6–0.8) that was supplemented with 20 mg acetosyringone/L for 15–20 min. Transgenic tobacco plants were generated on selection media after a 24 h light treatment following Wang’s method [22]. 

### 4.10. PCR Verification of Transformed PpNAC187 Tobacco

DNA was extracted from tobacco leaf tissue while using DNAplant Reagent (TianGen, Shanghai, China), according to the manufacturer’s instructions. *PpNAC187* primers listed in Table S2 were used to verify the presence of *PpNAC187*. The PCR program utilized was: 94 °C for 5 min., 35 cycles of 94 °C for 30 s, 60 °C for 1 min, and 72 °C for 1 min, followed by a 10 min. extension at 72 °C and a final cycle at 4 °C.

### 4.11. Wiesner Staining and Microscopy

Wiesner reagent (phloroglucinol/HCl) staining of plant tissue for 5 min was used to visualize lignification [5]. Two grams of phloroglucinol were dissolved in 100 mL of 95% alcohol and then filtered into 40 mL of concentrated hydrochloric acid. A razor blade was used to dissect the leaf tissue prior to observation. Lignified structures appeared pink or fuchsia in color. Auto-fluorescence within the stem sections was observed with the aid of an EVOS smart fluorescence microscope (Thermo Fisher, Waltham, MA, USA).

### 4.12. Lignin Assay 

Lignin content was assayed while using the method that was described by Bruce, with some modifications [38]. The samples were washed three times in a 10 mL solution (100 mM K2HPO4/KH2PO4, 0.5%Triton X-100, 0.5% PVP, PH 7.8), followed by an additional three washes in 100% methanol. The samples of fruit tissues were then dried overnight and the tissue samples were then transferred into 1 mL of solution that was composed of 2 M HCl and 0.1 mL thioglycolic acid. Lignin was extracted in this solution by placing the samples in a boiling water bath for 4 h. Pellets that were obtained by centrifugation were resuspended 2 mL 1M NaOH, followed by agitation for 18 h. The mixture was incubated for 4 h at 4 °C after the addition of 0.2 mL HCl. The end product was dissolved in 1 mL 1M NaOH and absorbance at 280 nm was recorded to estimate the lignin content. All of the measurements were performed in triplicate.

### 4.13. Statistical Analyses 

Two-tailed *t*-test and Duncan’s multiple-range test were performed to determine the statistical significance of differences between the samples. Figures were drawn while using Origin 6.0 (Microcal Software Inc. Northampton, MA, USA).

## 5. Conclusions

In the present study, we demonstrated that the expression of lignin biosynthesis-related genes, including *4CL*, *CCR*, *COMT*, and *CAD*, exhibited significantly increased in ‘Whangkeumbae’ pear ‘hard-end’ fruit. Furthermore, the transient overexpression of *PpNAC187* in ‘Whangkeumbae’ pear flesh induced the expression of lignin synthesis related genes *PpCCR* and *PpCOMT* and the degree of lignification. The lignin content in both stem and leaf of *PpNAC187*-overexpressing transgenic tobacco was increased. These results suggest that *PpNAC187* enhances lignin synthesis by regulating the expression of lignin synthesis related genes in ‘Whangkeumbae’ pear ‘hard-end’ fruit.

## Supplementary Materials

The following are available online. Figure S1: The morphology and lignin content of roots in PpNAC187-overexpressing transgenic tobacco plants. Table S1: Gene-specific primer sequences used in the RT-qPCR analysis of gene expression. Table S2: Primer sequences used in the cloning of *PpNAC187* and transgenic plant validation.

## References

[1] Charles F.P., Michael J.C., Lacy P.M., Dean H.R. (1971). Market Diseases of Apples, Pears, and Quince.

[2] Yamaguchi M., Kubo M., Fukuda H., Demura T. (2008). Vascular-related NACDOMAIN7 is involved in the differentiation of all types of xylem vessels in Arabidopsis roots and shoots. Plant J..

[3] Rose D.H., Mccolloch L.P., Fisher D.F. (1951). Market Diseases of Fruits and Vegetables: Apples, Pears, Quinces.

[4] Raese J., Drake S. (2006). Calcium foliar sprays for control of alfalfa greening, cork spot, and hard end in ‘anjou’ pears. J. Plant Nutr..

[5] Lu G.L., Li Z.J., Zhang X.F., Wang R., Yang S.L. (2015). Expression analysis of lignin associated genes in hard end pear (*Pyrus pyrifolia* Whangkeumbae) and its response to calcium chloride treatment conditions. J. Plant Growth Regul..

[6] Wang Y.L., Zhang X.F., Wang Y.Z., Yang S.L., Qu H.Y. (2018). The changes of intracellular calcium concentration and distribution in the hard end pear (*Pyrus pyrifolia* cv. ‘Whangkeumbae’) fruit. Cell Calcium.

[7] Yamamoto T., Watanabe S. (1982). Initial time of development of hard end disorder in ‘Bartlett’ pear. J. Japan Soc. Hort. Sci..

[8] Fumio T. (2012). Recent advances in research on Japanese pear rootstocks. J. Japan. Soc. Hort. Sci..

[9] Zhao Q., Dixon R.A. (2011). Transcriptional networks for lignin biosynthesis: More complex than we thought. Trends Plant Sci..

[10] Cheng X., Li M.L., Li D.H., Zhang J.Y., Jin Q., Sheng L.L., Cai Y.P., Lin Y. (2017). Characterization and analysis of *CCR* and *CAD* gene families at the whole-genome level for lignin synthesis of stone cells in pear (*Pyrus bretschneideri*) fruit. Biol. Open..

[11] Vermerris W., Abril A. (2015). Enhancing cellulose utilization for fuels and chemicals by genetic modification of plant cell wall architecture. Curr. Opin. Biotech..

[12] Wang W.Q., Zhang J., Ge H., Li S.J., Li X., Yin X.R., Grierson D., Chen K.S. (2016). *EjMYB8* transcriptionally regulates flesh lignification in loquat fruit. PLoS ONE.

[13] Akiyoshi K., Pulla K., Kazuya Y., Saori E., Keiko Y., Hiroyasu E. (2000). Functional analysis of tobacco LIM protein Ntlim1 involved in lignin biosynthesis. Plant J..

[14] Cassan-Wang H., Goué N., Saidi M.N., Legay S., Sivadon P., Goffner D., Grima-Pettenati J. (2013). Identification of novel transcription factors regulating secondary cell wall formation in *Arabidopsis*. Front. Plant Sci..

[15] Xu Q., Wang W.Q., Zeng J.K., Zhang J., Donald G., Li X., Yin X.R., Chen K.S. (2015). A NAC transcription factor, EjNAC1, affects lignification of loquat fruit by regulating lignin. Postharvest Biol. Tec..

[16] Vroemen C.W., Mordhorst A.P., Albrecht C., Kwaaitaal M.A., deVries S.C. (2003). The *CUP-SHAPED COTYLEDON3* gene is required for boundary and shoots meristem formation in *Arabidopsis*. Plant Cell.

[17] Mitsuda N., Iwase A., Yamamoto H., Yoshida M., Seki M., Shinozaki K., Ohme-Takagi M. (2007). NAC transcription factors, NST1 and NST3, are key regulators of the formation of secondary walls in woody tissues of Arabidopsis. Plant Cell.

[18] Zhong R.Q., Ye Z.H. (2007). Regulation of cell wall biosynthesis. Curr. Opin. Plant Biol..

[19] Zhong R.Q., Ye Z.H. (2009). Transcriptional regulation of lignin biosynthesis. Plant Signal. Behav..

[20] Ge H., Zhang J., Zhang Y.J., Li X., Yin X.R., Grierson D., Chen K.S. (2017). *EjNAC3* transcriptionally regulates chilling-induced lignification of loquat fruit via physical interaction with an atypical *CAD-like* gene. J. Exp. Bot..

[21] Ahmad M., Yan X.H., Li J.Z., Yang Q.S., Jamil W., Teng Y.W., Bai S.L. (2018). Genome wide identification and predicted functional analyses of NAC transcription factors in Asian pears. BMC Plant Biol..

[22] Wang Y.L., Zhang X.F., Yang S.L., Wang C.H., Lu G.L., Wang R., Yang Y.J., Li D.L. (2017). Heterogenous expression of *Pyrus pyrifolia PpCAD2* and *PpEXP2* in tobacco impacts lignin accumulation in transgenic plants. Gene.

[23] Olsen K.M., Lea U.S., Slimestad R., Verheul M., Lillo C. (2008). Differential expression of four *Arabidopsis PAL* genes; *PAL1* and *PAL2* have functional specialization in abiotic environmental-triggered flavonoid synthesis. J. Plant Physiol..

[24] Lee D., Meyer K., Chapple C., Douglas C.J. (1997). Antisense suppression of 4-coumarate: Coenzyme A ligase activity in *Arabidopsis* leads to altered lignin subunit composition. Plant Cell.

[25] Jean C.L., Rebecca D., Kris M., Vronique S., Catherine L., Brigitte P., Annette N. (2007). Downregulation of cinnamoyl-coenzyme A reductase in poplar: Multiple-level phenotyping reveals effects on cell wall polymer metabolism and structure. Plant Cell.

[26] Guo D., Chen F., Inoue K., Blount J.W., Dixon R.A. (2001). Downregulation of Caffeic Acid 3-O-Methyltransferase and Caffeoyl CoA 3-O-Methyltransferase in transgenic alfalfa: Impacts on lignin structure and implications for the biosynthesis of G and S lignin. Plant Cell.

[27] Liu W.S., Stewart C.N. (2016). Plant synthetic promoters and transcription factors. Curr. Opin. Biotech..

[28] Zhong R., Demura T., Ye Z.H. (2006). SND1, a NAC domain transcription factor, is a key regulator of secondary wall synthesis in fibers of *Arabidopsis*. Plant Cell.

[29] Kubo M., Udagawa M., Nishikubo N., Horiguchi G., Yamaguchi M., Ito J., Mimura T., Fukuda H., Demura T. (2005). Transcription switches for protoxylem and metaxylem vessel formation. Gene Dev..

[30] Trapnell C., Pachter L., Salzberg S.L. (2009). TopHat: discovering splice junctions with RNA-Seq. Bioinformatics.

[31] Wu T., Zhang R., Gu C., Wu J., Wan H., Zhang S., Zhang S. (2012). Evaluation of candidate reference genes for real time quantitative PCR normalization in pear fruit. Afr. J. Agric. Res..

[32] Niu S., Li Z., Yuan H., Fang P., Chen X., Li W. (2013). Proper gibberellin localization in vascular tissue is required to regulate adventitious root development in tobacco. J. Exp. Bot..

[33] Livak K.J., Schmittgen T.D. (2011). Analysis of relative gene expression data using real-time quantitative PCR and the 2^−ΔΔCt^ method. Method.

[34] Weigel D., Glazebrook J. (2006). Transformation of *Agrobacterium* using the freeze-thaw method. CSH Protoc..

[35] Sun W., Cao Z., Li Y., Zhao Y.X., Zhang H. (2007). A simple and effective method for protein subcellular localization using *Agrobacterium*-mediated transformation of onion epidermal cells. Biologia.

[36] Spolaore S., Casadoro G., Trainotti L. (2001). A simple protocol for transient gene expression in ripe fleshy fruit mediated by *Agrobacterium*. J. Exp. Bot..

[37] Zheng L., Liu G.F., Meng X.N., Li Y.B., Wang Y.C. (2012). A versatile *Agrobacterium*-mediated transient gene expression system for herbaceous plants and trees. Biochem. Genet..

[38] Bruce R.J., West C.A. (1989). Elicitation of lignin biosynthesis and isoperoxidase activity by pectic fragments in suspension cultures of castor bean. Plant Physiol..

