# Supplementary material for: PpNAC187 Enhances Lignin Synthesis in ‘Whangkeumbae’ Pear (Pyrus pyrifolia) ‘Hard-End’ Fruit
Source: Molecules. 2019 Nov 27;24(23):4338. doi: 10.3390/molecules24234338 (PMC6930614; doi:10.3390/molecules24234338)
Supplement: Supplementary file 1 [file molecules-24-04338-s001.pdf]

Supplementary materials

Table 1. Gene-specific primer sequences used in the RT-qPCR analysis of gene expression.

Gene name	Gene ID	Primer name	Primer sequence
PpNAC1	PCP044783	PpNAC1-F	5′-CCAAACATACCCACCAAGCA -3′
		PpNAC1-R	5′-TGAGCTACTTCCTTCGCCAA-3′
PpNAC2	PCP012487	PpNAC2-F	5′-TGACAACACCAGAAACAGCG-3′
		PpNAC2-R	5′-CCGAACTGAAACATTTGCGC-3′
PpNAC3	PCP013078	PpNAC3-F	5′-AAAGATGGTGAGCTGGTGGT-3′
		PpNAC3-R	5′-GTACTCCACAAGACCTGCCT-3′
PpCOMT	PCP007841	PpCOMT-F	5′-GTTTACACCGGCTACTCCCT -3′
		PpCOMT-R	5′-CCCCAACCTTCACCAAATCG -3′
Pp4CL	PCP024172	Pp4CL-F	5′-ACTCCTACTGCCTCCACAAC -3′
		Pp4CL-R	5′-GCTGCCTCATCCTTCATTGG -3′
PpCAD1	JX290376	PpCAD1-F	5′-GATGTCACAGACCCAAAGGCA-3′
		PpCAD1-R	5′-AGGCGTTCGAGGTTTTCCAT-3′
PpCAD2	KJ577637	PpCAD2-F	5′-TTTGGTTGAGAGAGTTGCCCAC-3′
		PpCAD2-R	5′-ATTCGACACCCAAGCTCTTCG-3′
PpCCR	PCP040222	PpCCR-F	5′- ATGTCGAAGCAGGGTGAGGT-3′
		PpCCR-R	5′- CTAGGCATTGGAGTAACTCA-3′
PcActin	AF386514	PcActin-F	5′-CCCAGAAGTGCTCTTCCAAC-3′
		PcActin-R	5′-TTGATCTTCATGCTGCTTGG-3′
NtActin	AB158612	NtActin-F	5′-TGTTGGAATGGAAGCTGCTG-3′

	NtActin-R	5′-TTTGCGGTGGACAATGGAAG-3′

Table 2. Primer sequences used in the cloning of *PpNAC187* and transgenic plant validation.

Gene name	Primer name	Primer sequence
PpNAC3	PpNAC3-F	5′-ATGTCTTCCTCCATGGCATCCTCAG -3′
PCP013078	PpNAC3-R	5′- TCACTTTGGTACTCCATCTACAACG-3′
PpNAC3 (XhoI HindIII) PCP013078	PpNAC3-F	5′-GCGCTCGAGATGTCTTCCTCCATGGCATCCTCAG -3′
	PpNAC3-R	5′- GCGAAGCTTTCACTTTGGTACTCCATCTACAACG-3′
PpNAC3 (KpnI HindIII) PCP013078	PpNAC3-F	5′-GCGAAGCTTATGTCTTCCTCCATGGCATCCTCAG -3′
	PpNAC3-R	5′- GCGGGTACCTCACTTTGGTACTCCATCTACAACG-3′
NPTII	NPTII-F	5′-ATGATTGAACAAGATGGATTGC -3′
	NPTII-R	5′-TCAGAAGAACTCGTCAAGAAGG -3′

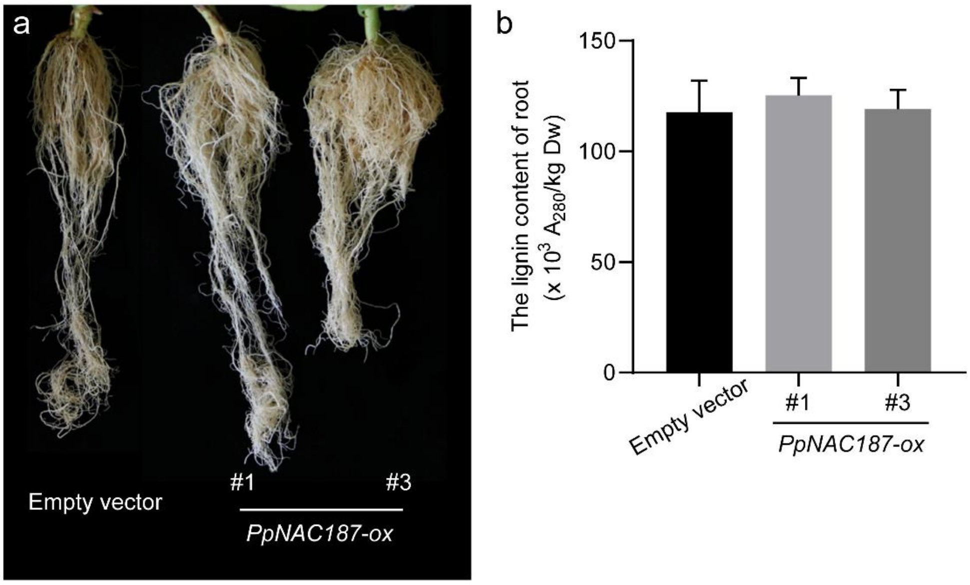

Figure S1 The morphology and lignin content of roots in *PpNAC187*-overexpressing transgenic tobacco plants.
